# Supplementary material for: Periodic synchronization of isolated network elements facilitates simulating and inferring gene regulatory networks including stochastic molecular kinetics
Source: BMC Bioinformatics. 2022 Jan 5;23:13. doi: 10.1186/s12859-021-04541-6 (PMC8729106; doi:10.1186/s12859-021-04541-6)
Supplement: Supplementary file 1 — Additional file 1: Table S1. Kinetic parameters of the repressive gene cascade (Figure 2 of the main text). [file 12859_2021_4541_MOESM1_ESM.pdf]

Additional Table 1: Kinetic parameters of the repressive gene cascade.

Parameter	Scenario: Slow Switching	Scenario: Fast Switching
Gene 1		
λ_A	0.05	1
μ_A	0.05	1
λ_R	4.4	87
μ_R	0.05	1
ν	0.1	0.1
δ	0.001	0.001
Gene 2		
λ_A	0.05	1
μ_A	0.05	1
λ_R	4.1	82
μ_R	0.05	1
ν	0.1	0.1
δ	0.001	0.001
Gene 3		
λ_A	0.05	1
μ_A	0.05	1
λ_R	12.5	250
μ_R	0.05	1
ν	0.1	0.1
δ	0.001	0.001
Gene 4		
λ_A	0.05	1
μ_A	0.05	1
λ_R	8	160
μ_R	0.05	1
ν	0.5	0.5
δ	1e-3	1e-3
