# Supplementary material for: Periodic synchronization of isolated network elements facilitates simulating and inferring gene regulatory networks including stochastic molecular kinetics
Source: BMC Bioinformatics. 2022 Jan 5;23:13. doi: 10.1186/s12859-021-04541-6 (PMC8729106; doi:10.1186/s12859-021-04541-6)
Supplement: Supplementary file 2 — Additional file 2: Table S2. Kinetic parameters of the exemplary biochemical reaction (Figure 4 of the main text). [file 12859_2021_4541_MOESM2_ESM.pdf]

Additional Table 2: Kinetic parameters of the exemplary biochemical reaction (Figure 4 of the main text).

Parameter	Values
Enzyme 1	
λ	0.001
μ	0.1
ν	0.2
δ	0.001
Enzyme 2	
λ	0.001
μ	0.1
ν	0.1
δ	0.001
