# Supplementary material for: Periodic synchronization of isolated network elements facilitates simulating and inferring gene regulatory networks including stochastic molecular kinetics
Source: BMC Bioinformatics. 2022 Jan 5;23:13. doi: 10.1186/s12859-021-04541-6 (PMC8729106; doi:10.1186/s12859-021-04541-6)
Supplement: Supplementary file 3 — Additional file 3: Table S3. Kinetic parameters of the heterogeneous GRNs (Figure 5 of the main text). [file 12859_2021_4541_MOESM3_ESM.pdf]

Additional Table 3: Kinetic parameters of the heterogeneous GRNs (Figure 5 of the main text).

Parameters of the positive feedback loop	Value [s^{-1}]
λ_A	2e-4
μ_A	0.01
ν	0.2
β	10
λ_E	3.3e-4
μ_E	3.3e-3
ν_E	1e-3
δ	1e-3
Total number of Enzymes	45

Parameters of the negative feedback loop	Value [s^{-1}]
λ_R	0.04
μ_R	0.01
ν	10.2
β	1e-5
λ_E	0.0022
μ_E	1e-4
ν_E	1e-3
δ	1e-9
Total number of Enzymes	50
