# Supplementary material for: Periodic synchronization of isolated network elements facilitates simulating and inferring gene regulatory networks including stochastic molecular kinetics
Source: BMC Bioinformatics. 2022 Jan 5;23:13. doi: 10.1186/s12859-021-04541-6 (PMC8729106; doi:10.1186/s12859-021-04541-6)
Supplement: Supplementary file 4 — Additional file 4: Table S4. Kinetic parameters of the exemplary GRN used to demonstrate the network inference (Figure 6 of the main text). [file 12859_2021_4541_MOESM4_ESM.pdf]

Additional Table 4: Kinetic parameters of the exemplary GRN used to demonstrate the network inference (Figure 6 of the main text).

Parameter	Scenario: Slow Switching	Equilibrium constant
Gene 1		
λ_A	0.01	0.1
μ_A	0.1	
λ_R	0.01	0.1
μ_R	0.1	
ν	0.1	
δ	0.001	
Gene 2		
λ_A	0.01	0.1
μ_A	0.1	
ν	0.1	
δ	0.001	
