# Supplementary material for: Periodic synchronization of isolated network elements facilitates simulating and inferring gene regulatory networks including stochastic molecular kinetics
Source: BMC Bioinformatics. 2022 Jan 5;23:13. doi: 10.1186/s12859-021-04541-6 (PMC8729106; doi:10.1186/s12859-021-04541-6)
Supplement: Supplementary file 5 — Additional file 5: Table S5. Number of inferred true and false positive and true and false negative connections for networks of N = 5 to 10 genes as result of the network inference process (Figure 6 of the main text). [file 12859_2021_4541_MOESM5_ESM.pdf]

Additional Table 5: Number of inferred true and false positive and true and false negative connections for networks of N=5 to 10 genes as result of the network inference process (Figure 6 of the main text).

True Positive	False Negative	False Positive	True Negative	True Positive Rate	False Positive Rate
5 Genes					
16	2	2	10	0,89	0,17
20	0	1	9	1,00	0,10
17	1	2	10	0,94	0,17
15	0	1	14	1,00	0,07
14	2	4	10	0,88	0,29
14	1	10	5	0,93	0,67
17	0	2	11	1,00	0,15
19	0	2	9	1,00	0,18
12	1	4	13	0,92	0,24
13	1	3	13	0,93	0,19
18	1	2	9	0,95	0,18
17	1	2	10	0,94	0,17
6 Genes					
26	1	2	13	0,96	0,13
28	0	2	12	1,00	0,14
24	1	4	13	0,96	0,24
22	0	3	17	1,00	0,15
19	2	12	9	0,90	0,57
29	0	0	13	1,00	0,00
25	3	3	11	0,89	0,21
24	0	0	18	1,00	0,00
22	2	9	9	0,92	0,50
22	1	4	15	0,96	0,21
22	3	4	13	0,88	0,24
29	0	2	11	1,00	0,15
7 Genes					
29	0	2	25	1,00	0,07
26	0	3	27	1,00	0,10
24	1	6	25	0,96	0,19
28	2	12	14	0,93	0,46
30	1	1	24	0,97	0,04
29	0	11	16	1,00	0,41
18	13	12	13	0,58	0,48
29	0	1	26	1,00	0,04
31	0	5	20	1,00	0,20
19	2	11	24	0,90	0,31
30	1	2	23	0,97	0,08
27	1	1	27	0,96	0,04
8 Genes					
39	1	5	27	0,98	0,16
28	13	12	19	0,68	0,39
32	8	13	19	0,80	0,41
44	0	2	26	1,00	0,07
34	3	7	28	0,92	0,20
38	0	3	31	1,00	0,09
37	4	11	20	0,90	0,35
35	3	8	26	0,92	0,24
35	0	0	37	1,00	0,00
46	0	5	21	1,00	0,19
9 Genes					
49	0	2	39	1,00	0,05
39	3	18	30	0,93	0,38
51	0	1	38	1,00	0,03
44	7	17	22	0,86	0,44
35	8	12	35	0,81	0,26
50	1	2	37	0,98	0,05
49	1	3	37	0,98	0,08
47	1	3	39	0,98	0,07
50	1	21	18	0,98	0,54
54	0	10	26	1,00	0,28
10 Genes					
62	0	3	45	1,00	0,06
66	0	21	23	1,00	0,48
55	0	0	55	1,00	0,00
60	0	7	43	1,00	0,14

62	1	11	36	0,98	0,23
55	1	3	51	0,98	0,06
54	0	3	53	1,00	0,05
59	0	1	50	1,00	0,02
55	0	4	51	1,00	0,07
56	1	6	47	0,98	0,11
